# Identification of Prognostic miRNAs Associated With Immune Cell Tumor Infiltration Predictive of Clinical Outcomes in Patients With Non-Small Cell Lung Cancer

**DOI:** 10.3389/fonc.2021.705869

**Published:** 2021-07-01

**Authors:** Yuepeng Zhang, Kai Mi, Zhiheng Li, Lixia Qiang, Meiyu Lv, Yushan Wu, Ligong Yuan, Shoude Jin

**Affiliations:** ^1^ Department of Respiratory, The Fourth Affiliated Hospital of Harbin Medical University, Harbin, China; ^2^ College of Bioinformatics Science and Technology, Harbin Medical University, Harbin, China; ^3^ Department of Medical Oncology, The Fourth Affiliated Hospital of Harbin Medical University, Harbin, China; ^4^ Department of Thoracic Surgery, National Cancer Center/National Clinical Research Center for Cancer/Cancer Hospital, Chinese Academy of Medical Sciences & Peking Union Medical College, Beijing, China

**Keywords:** NSCLC, immune infiltration, miRNAs, prognosis, miRNA-target network

## Abstract

**Background:**

A detailed means of prognostic stratification in patients with non-small cell lung cancer (NSCLC) is urgently needed to support individualized treatment plans. Recently, microRNAs (miRNAs) have been used as biomarkers due to their previously reported prognostic roles in cancer. This study aimed to construct an immune-related miRNA signature that effectively predicts NSCLC patient prognosis.

**Methods:**

The miRNAs and mRNA expression and mutation data of NSCLC was obtained from The Cancer Genome Atlas (TCGA). Immune-associated miRNAs were identified using immune scores calculated by the ESTIMATE algorithm. LASSO-penalized multivariate survival models were using for development of a tumor immune-related miRNA signature (TIM-Sig), which was evaluated in several public cohorts from the Gene Expression Omnibus (GEO) and the CellMiner database. The miRTarBase was used for constructing the miRNA-target interactions.

**Results:**

The TIM-Sig, including 10 immune-related miRNAs, was constructed and successfully predicted overall survival (OS) in the validation cohorts. TIM-Sig score negatively correlated with CD8+ T cell infiltration, IFN-γ expression, CYT activity, and tumor mutation burden. The correlation between TIM-Sig score and genomic mutation and cancer chemotherapeutics was also evaluated. A miRNA-target network of 10 miRNAs in TIM-Sig was constructed. Further analysis revealed that these target genes showed prognostic value in both lung squamous cell carcinoma and adenocarcinoma.

**Conclusions:**

We concluded that the immune-related miRNAs demonstrated a potential value in clinical prognosis.

## Introduction

The most malignant and most commonly encountered lung cancer subtype is non-small cell lung cancer (NSCLC) (1). The NSCLC subtype can further be classified as either lung adenocarcinoma (LUAD) or lung squamous cell carcinoma (LUSC). Forty percent of all lung cancers are of the LUAD subtype, with LUSC reported as the second leading cause of lung malignancy-related death, resulting in an average of 400,000 deaths worldwide annually (2). Although several new treatment regimens including chemotherapeutic and biological agents have been introduced, the effectiveness of these protocols have been marred by the occurrence of drug resistance, leading to inevitably poor outcomes for patients with advanced NSCLC (3). Immune-directed therapy has, in recent years, shown better efficacy and lower toxicity rates over regular chemotherapy in NSCLC. Nevertheless, durable benefits from immunotherapy are reported in only 25–30% of patients (4). Therefore, more effective prognostic biomarkers for risk stratification in NSCLC are required.

MicroRNAs (miRNAs) represent long, non-coding RNAs of approximately 22 nucleotides in length. These molecules are central in posttranscriptional regulation (5). Both tumor initiation and metastasis have been reported to depend heavily on miRNA expression, with certain miRNAs shown to be associated with poor outcomes in NSCLC (6). A myriad of immune-related processes such as the development, activation, and effector functions of various innate and adaptive immune cells have been linked to miRNAs, which therefore appear to be directly responsible in regulating the infiltration of immune cells into tumors (7, 8). Growing evidence has depicted the key function of the tumor-infiltrating immune cell (TIIC) in tumor progression and prognosis (9, 10). Signatures associated with TIIC show promising predictive values in prognosis and responses to immunotherapy in patients with NSCLC (11, 12). Previous research showed that these signatures may be obtained by exploring the expression of certain miRNAs. In cervical cancer, miR-1468-5p was found to upregulate lymphatic PD-L1 and augment lymphangiogenesis, both of which result in dysregulated T cell immunity (13). Reduced miR-4772-3p levels were inversely related to the concentration of Tregs in malignant pleural effusion (MPE) (8). Furthermore, the miR141-CXCL1-CXCR2 pathway was found to modulate Tregs migration into MPE (7). Nevertheless, these studies were on single miRNAs only. An integrated model comprising of a variety of biomarkers has been shown to offer higher predictive capabilities in comparison to models of single biomarkers (14). Construction of multiple biomarker models using conventional Cox regression models has been problematic and often suffers from high rates of model overfitting especially in the context of a large number of biomarkers. The least absolute shrinkage and selection operator (LASSO)-penalized Cox model has been introduced to implement variable selection and has been applied successfully for creating models of several biomarkers (15). This study uses the LASSO technique to construct a multi-miRNA-based signature to provide an immune infiltration score (TIM-Sig score) which is able to stratify NSCLS patients according to their prognosis. We further systematically correlated the TIM-Sig score with available genetic and clinical features of NSCLC patients.

## Materials and Methods

### Dataset Preprocessing

Transcriptional profiles and clinical information for lung cancer were obtained from the Gene Expression Omnibus (GEO, http://www.ncbi.nlm.nih.gov/geo) and The Cancer Genome Atlas (TCGA, https://portal.gdc.cancer.gov). The miRNA expression profiles were obtained from the UCSC Xena browser (GDC hub: https:/gdc.xenahubs.net). A total of 1,884 miRNAs were obtained for the following analysis. Five of the following NSCLC cohorts were also processed by log2 transformation: the TCGA-LUAD/LUSC cohort, GSE16025 cohort, GSE27435 cohort, GSE31210 cohort, and GSE3141 cohort. A brief summary of the clinical and pathological characteristics is shown in **Table 1**. We also downloaded somatic mutation data from TCGA as calculated by the mutect2 workflow.

**Table 1:** Clinicopathological characteristics of NSCLC patients in this study.

Characteristics Platform	Training series	Testing series		GSE31210 HG-U133_Plus_2	GSE3141 HG-U133_Plus_2
	TCGA IlluminaHiSeq	GSE16025 GPL5106	GSE27435 GPL8469		
Patients	1014	71	42	246	111
>60	721	51	21	124	NA
≤60	265	20	21	117	NA
NA	28	0	0	5	NA
Gender					
Female	406	49	12	130	NA
Male	608	22	30	116	NA
Stage					
I	518	42	NA	168	NA
II	283	16	NA	58	NA
III	168	13	NA	0	NA
IV	33	0	NA	0	NA
NA	12	0	NA	20	NA
Survival					
Dead	284	35	18	35	54
Alive	730	26	24	191	57
NA	0	0	0	20	0

NA, no information.

### LASSO Mixture and Cox Regression Models for Predicting Survival

Tumor purity and tumor immune scores were derived using the ESTIMATE algorithm which is a novel algorithm by Yoshihara et al. It is a method using gene expression profiles to evaluate the fraction of stromal and immune cells in tumor samples. The ESTIMATE algorithm generates three scores: stromal score, immune score, and estimate score (16). The immune score was used to selected immune-related miRNAs. The Spearman correlation coefficient between differentially expressed miRNAs (DEMs) and the immune score was calculated with significance set at (|R| > 0.2, P < 0.01). A total of 35 miRNAs significantly correlated with the immune score were identified and analyzed in the LASSO regression model. The R package “glmnet” and “survival” were used to carry out LASSO and Cox regression analyses to assess the relationship between overall NSCLS patient survival and DEG expression levels. We identified a tumor-infiltrating immune-related miRNA signature score (TIM-Sig score) with the following formula:

$$TIM-sig\;score=\sum_{1}^{n}coef_{i}\times EXP_{I}$$

in which *n* represents the total number of prognostic miRNAs or genes, *EXP_I_* represents *gene_i_* profile expression and *coef_i_* represents an estimate of the *gene_i_* regression coefficient as identified using the multivariable Cox regression analysis or LASSO.

### Approximation of Tumor-Infiltrating Immune Cells

The proportion of immune cell infiltration was estimated using the “GSVA” R package and 27 human immune cell phenotypes (17–19). In addition, factors related to tumor immunogenicity were also contrasted between high- and low-risk groups. These factors are as follows: tumor mutation burden (TMB) (17), IFN-γ expression signature (20), chromosomal instability level (HRD) (21), immune cytotoxic activity (CYT) (22–24), T cell infiltration score (TIS) (20, 25), relative antigen presentation machinery (APM) (19), and tumor-infiltrating lymphocytes (TILs) (26–29). All these factors were selected based on the status of specific biomarker genes, such as the presence of costimulatory factors or major histocompatibility complex (MHC) molecules.

### Assessment of the Clinical Significance of the miRNA Signature

To determine the value of the constructed model in the clinical management of patients with lung cancer, miRNA profiles and drug sensitivity IC50 values of the NCI-60 panel of human cancer cell lines were extracted from the CellMiner database (https://discover.nci.nih.gov/cellminer/) (30). The therapeutic effects of 161 Food and Drug Administration (FDA)-approved drugs in NSCLC patients were determined. The Wilcoxon test was used to analyze the significance between differences in the IC50 Z-score between the high- and low-risk cohorts. Results are depicted in terms of box drawings plotted using the ggplot2 function of R.

### MiRNA-Target Interactions

The miRTarBase (http://mirtarbase.cuhk.edu.cn/php/index.php) is a database containing over 430,000 miRNA-target interactions (MTIs) (31). All documented MTIs have been verified using next-generation sequencing, microarray, western blot, and reporter assay experiments. We obtained the target information of 10 miRNA in TIM-Sig to construct the miRNA-target network.

### Functional Analysis

Kyoto Encyclopedia of Genes and Genomes (KEGG) pathway analysis was performed using the Database for Annotation, Visualization, and Integrated Discovery (DAVID) tool. We obtained pathway function annotations of 1,862 target genes. The statistical threshold was set as: P < 0.05.

### Statistical Analysis

The R software (version 3.6.1, http://www.R-project.org) was used to derive all statistical analyses. Differentially expressed miRNAs or genes were calculated using R packages “DESeq2.” Spearman correlation between signatures were calculated by the corr. test () function in the R program. Overall survival (OS) was predicted using Kaplan-Meier survival plots. The R package “survival” function was used to assess for differences between the OS of high- and low-groups, with a p-value of less than 0.05 taken to indicate statistical significance. The Wilcoxon test allowed for inter-group comparisons. Euclidean distances and Ward’s linkage methods were used to carry out hierarchical cluster analyses. Protein-protein interaction (PPI) networks were visualized on STRING tools.

### MiRNA-Targets Network

The construction of miRNA-target networks and parameter settings were completed using Cytoscape tools (version 3.6.0). A summary and oncoplot of mutation data were calculated using R package “maftools.” All statistical tests with a p-value of less than 0.05 were identified as having achieved statistical significance (*p-value < 0.05; **p-value < 0.005; ***p-value < 0.0005; ****p-value < 0.00005).

## Results

### Screening of Candidate Immune-Related miRNAs

**Figure 1:**
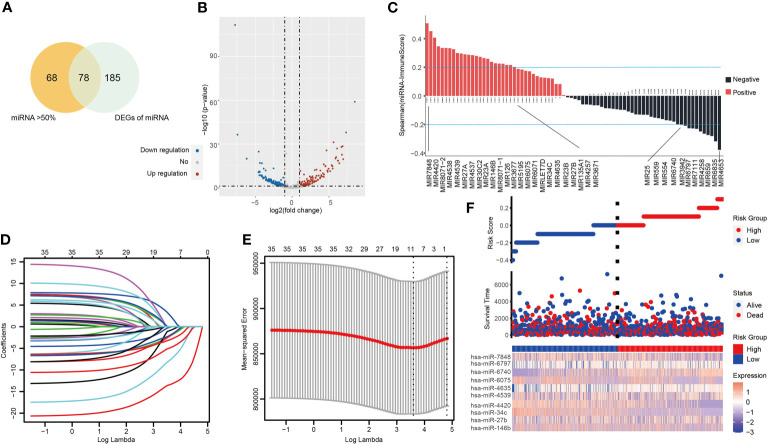
The tumor-infiltrating immune-related miRNA signature (TIM-Sig). **(A)** miRNAs expressed in more than 50% of the tumor samples and are differentially expressed. **(B)** Volcano plot of 263 identified differential expression miRNAs. The cutoffs were set as a log2(fold-change) > 1.0 or < –1.0 and p-value < 0.05. **(C)** miRNAs expression associated with immune score, as shown by Spearman correlation analysis. **(D, E)** The LASSO regression method was carried out to identify the 10 critical miRNAs associated with tumor immune infiltration. **(F)** The log-rank test and univariate Cox analysis were used to process data. Patterns of miRNAs expression and proportion of high-and low-risk patients in the verification dataset.

**Figure S1** demonstrates the workflow of this study. In order to screen potential tumor immune-related miRNA biomarkers in NSCLC, a total of 1,884 miRNAs were obtained from the TCGA LUAD/LUSC cohort. Next, we filtered the miRNAs according to the following selection criteria: (1) positive expression in more than 50% of samples; (2) are differentially expressed, with a statistical threshold of |*log_2_FC*| > 1 and p-value <0.05, including 263 DEMs (153 downregulated and 110 unregulated). Seventy-eight overlapping miRNAs were selected as candidates for subsequent analysis (**Figures 1A, B** and **Figure S2A**). The ESTIMATE algorithm was used to determine tumor purity and tumor immune scores. Spearman correlation analysis was then applied to assess the relationship between the 78 miRNAs and immune score, resulting in a total of 35 miRNAs which were significantly associated with the immune scores (|Spearman correlation| > 0.2 and P < 0.01) (**Figure 1C** and **Table S1**). Interestingly, we found that 15 miRNAs were significantly positively correlated to the stromal score also correlated positively to the immune scores. All of these miRNAs were negatively associated with tumor purity (**Figure 1C** and **Figures S2B, C**).

A LASSO proportional hazards regression analysis was then performed to determine the relationship between patient OS and the expressions of the identified 35 miRNAs in the training data set (**Figures 1D, E**). We found a significant correlation with the OS of NSCLC patients with the following 10 miRNAs (hsa-miR-146b, hsa-miR-27b, hsa-miR-34c, hsa-miR-4420, hsa-miR-4539, hsa-miR-4635, hsa-miR-6075, hsa-miR-6740, hsa-miR-6797, and hsa-miR-7848) (**Table S2**). These markers were considered to function as prognostically significant immune-related miRNAs. We then implemented these 10 miRNAs in the development of a miRNA-based prognostic score that determined the degree of immune tumor infiltration (TIM-Sig score). These 10 miRNAs were weighted based on the LASSO regression coefficient as follows: TIM-Sig score = (0.2899*expression value of hsa-miR-146b) + (0.2789*expression value of hsa-miR-27b) + (0.4310*expression value of hsa-miR-34c) + (0.7648*expression value of hsa-miR-4420) + (−0.3621*expression value of hsa-miR-7848) + (−0.3577*expression value of hsa-miR-4635) + (0.0819*expression value of hsa-miR-6075) + (0.3234*expression value of hsa-miR-6740) + (−0.0322*expression value of hsa-miR-6797) + (0.5820*expression value of hsa-miR-4539). The distribution of TIM-Sig score and expression pattern of miRNAs are revealed in **Figure 1F**.

### Prognostic Value of TIM-Sig

**Figure 2:**
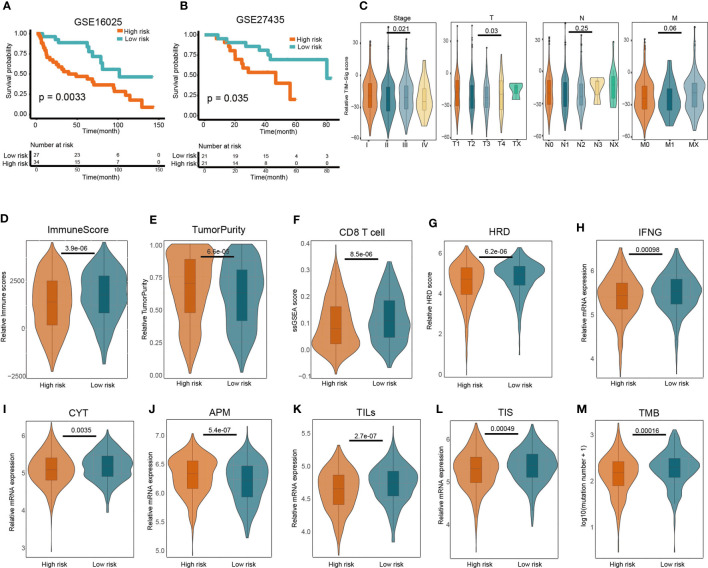
Predictive value of the 10-miRNA signature in NSCLC patients and heterogeneous immune infiltration in high- and low-risk groups. **(A, B)** Kaplan–Meier predictions of patient overall survival in those of low- or high-risk as stratified using the TIM-Sig (GSE16025 and GSE27435). The log-rank test was used to carry out statistical analysis. **(C)** The difference of TIM-Sig score in clinical classification (include stage and TNM classification). **(D)** Relative immune scores between low- and high-risk cohorts. **(E)** Comparison of relative tumor purity between low- and high-risk cohorts. **(F)** Comparison of relative CD8 T cell score based on ssGSEA between low- and high-risk cohorts. **(G)** Comparison of relative chromosomal instability level between low- and high-risk cohorts. **(H)** Comparison of relative IFN-γ expression signature between low- and high-risk cohorts. **(I)** Comparison of relative cytotoxic activity scores between low- and high-risk cohorts. **(J)** Comparison of relative antigen presentation machinery between low- and high-risk cohorts. **(K)** Comparison of relative tumor-infiltrating lymphocytes between low- and high-risk cohorts. **(L)** Comparison of relative T cell infiltration score between low- and high-risk cohorts. **(M)** Comparison of relative tumor mutation burden between low- and high-risk cohorts. The p-values were calculated using the Wilcoxon test.

The GSE27435 and GSE16025 cohorts were used to verify our constructed TIM-Sig. The TIM-Sig score was determined for all subjects in the cohort, with the median values used to stratify patients as being either high- or low-risk. Patients with higher risk scores were noted to have poorer OS in contrast to those with lower risk scores (GSE16025: P = 0.0033; GSE27435: P = 0.035), as depicted in **Figures 2A, B**. The clinical value of the constructed 10-miRNA signature in prognosticating patients with lung cancer was verified.

### Potential of the TIM-Sig as an Indicator of Immune and Clinical Factors

We next investigated whether the TIM-Sig score was associated with tumor TNM classification or patient gender. We found a significant difference of TIM-Sig score with tumor size, distant metastasis as well as stage (Wilcoxon test, p-value <0.05; **Figure 2C** and **Figure S3**). The difference of some immune factors such as the CYT activity, APM score, TILs score, TIS score, chromosomal instability level, tumor mutation burden, IFN-γ expression signature, and T cell infiltration score (TIS) between TIM-Sig high- and low-risk groups were also assessed. A higher immune score (Wilcoxon test, p-value = 3.9e-06; **Figure 2D**), CD8 T cell score (Wilcoxon test, p-value = 8.5e-06; **Figure 2F**), HRD (Wilcoxon test, p-value = 6.02e-06; **Figure 2G**), IFN-γ expression signature (Wilcoxon test, p-value = 0.00098; **Figure 2H**), CYT activity (Wilcoxon test, p-value = 0.0035; **Figure 2I**), TILs score (Wilcoxon test, p-value = 2.7e-07; **Figure 2K**), TIS score (Wilcoxon test, p-value = 0.00049; **Figure 2L**) and TMB (Wilcoxon test, p-value = 0.00016; **Figure 2M**) were observed in the low-risk group of NSCLC patients. On the contrary, the higher value of tumor purity (Wilcoxon test, p-value = 6.6e-05; **Figure 2E**) and APM (Wilcoxon test, p-value = 5.4e-07; **Figure 2J**) were found in high risk group. Generally, these immune factors varied significantly between high- and low-risk groups. Furthermore, we also investigated the correlation between the TIM-Sig score and above immune-related factors. We found that the majority of these immune factors were negatively correlated with the TIM-Sig score (**Figure S4**). Based on these results, we conclude that there exists a close relationship between TIM-Sig and immune infiltration as well as the immune escape mechanism.

### Relationship Between Mutation and TIM-Sig

**Figure 3:**
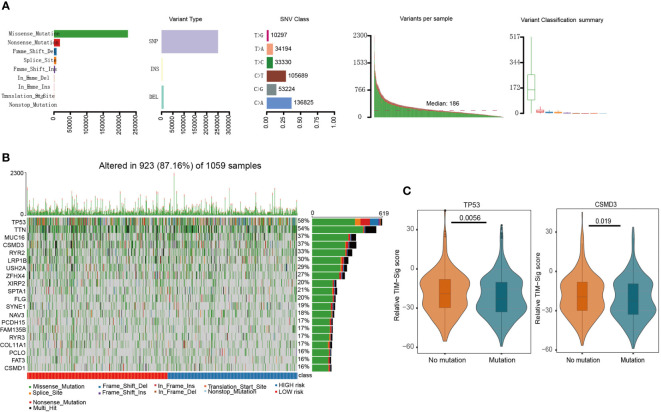
Mutations in NSCLC samples. **(A)** We utilized the maftools package to visualize the mutational features in NSCLC. In summary, we classified these mutation data into different categories, where missense mutation occupied the most part, single nucleotide polymorphism (SNP) mutated the most frequently, and C>A was the top type of single nucleotide variants (SNV) in NSCLC. **(B)** We exhibited the top 20 mutated genes, including well-known TP53 and MUC16. **(C)** The difference of TIM-Sig score in mutation and wild groups of TP53 and CSMD3. The p-values were calculated using the Wilcoxon test.

**Figure 3A** depicts identified somatic mutations in LUAD and LUSC patients. TP53 mutations were found in 58% of samples based on TCGA data, which depict the top 20 most frequently encountered gene mutations in lung cancer (**Figure 3B**). In addition, we compared the TIM-Sig score between the mutant and wild samples. Both groups appeared to differ significantly in terms of frequency of TP53 and CSMD3 mutations (Wilcoxon test, p-value = 0.0056 and 0.019 for TP53 and CSMD3 groups, respectively; **Figure 3C**). The frequency that each mutation was encountered also varied between high- and low-risk groups (**Figure S5**).

### TIM-Sig Could Predict Chemotherapeutics Response

**Figure 4:**
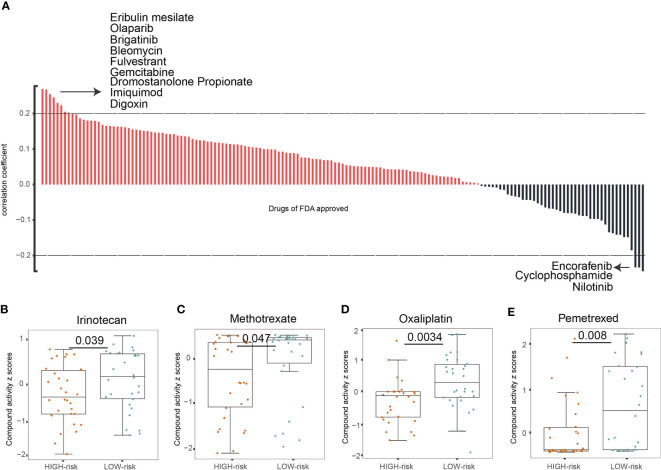
The TIM-Sig model as a potential predictor for chemosensitivity. **(A)** The respective IC50 value of chosen compounds in relation to the TIM-Sig score, as shown by Spearman correlation analysis. **(B–E)** Those with high risk-scores were found to possess lower IC50 scores for FDA-approved chemotherapeutics such as Irinotecan, Methotrexate, Oxaliplatin, and Pemetrexed. The p-values were calculated using the Wilcoxon test.

We then investigated whether the TIM-Sig could predict chemosensitivity. To do this, we calculated the TIM-Sig score of NCI60 cell lines using the expression data available in a cellminer database (60 cell lines). The association between the TIM-Sig score and the inhibitory centration (*IC*_50_) value of 161 FDA-approved drugs across 60 cell lines were calculated. The result showed that Eribulin mesylate, Olaparib, Brigatinib, Bleomycin, Fulvestrant, Gemcitabine, Dromostanolone Propionate, Imiquimod, and Digoxin appeared to correlate significantly with the risk model (|Spearman correlation| > 0.2 and p < 0.01, **Figure 4A**). A high immune score was linked to a lower half inhibitory centration (*IC*_50_) of medications including Irinotecan (Wilcoxon test, p = 0.039, **Figure 4B**), Methotrexate (Wilcoxon test, p < 0.047, **Figure 4C**), Oxaliplatin (Wilcoxon test, p < 0.0034, **Figure 4D**), and Pemetrexed (Wilcoxon test, p < 0.008, **Figure 4E**). These findings suggest that the model was able to function as a chemosensitivity predictor.

### Identification of TIM-Sig Regulated Targets

**Figure 5:**
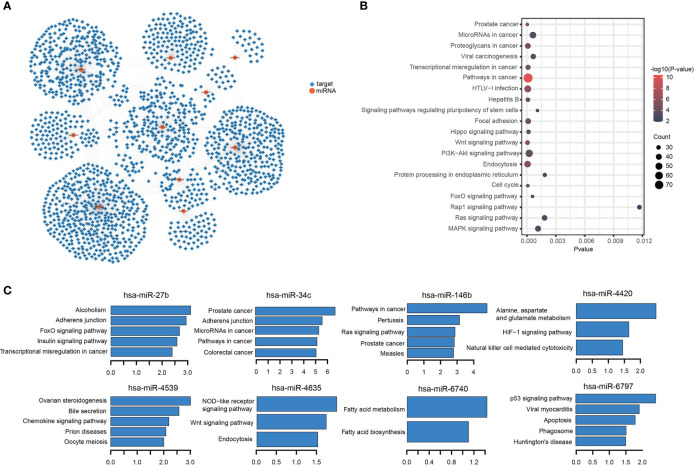
miRNAs-targets network and KEGG enrichment analysis. **(A)** miRNAs-targets network. Circular node denotes miRNAs, square node denotes targets. **(B, C)** Results for KEGG enrichment analysis.

**Figure 6:**
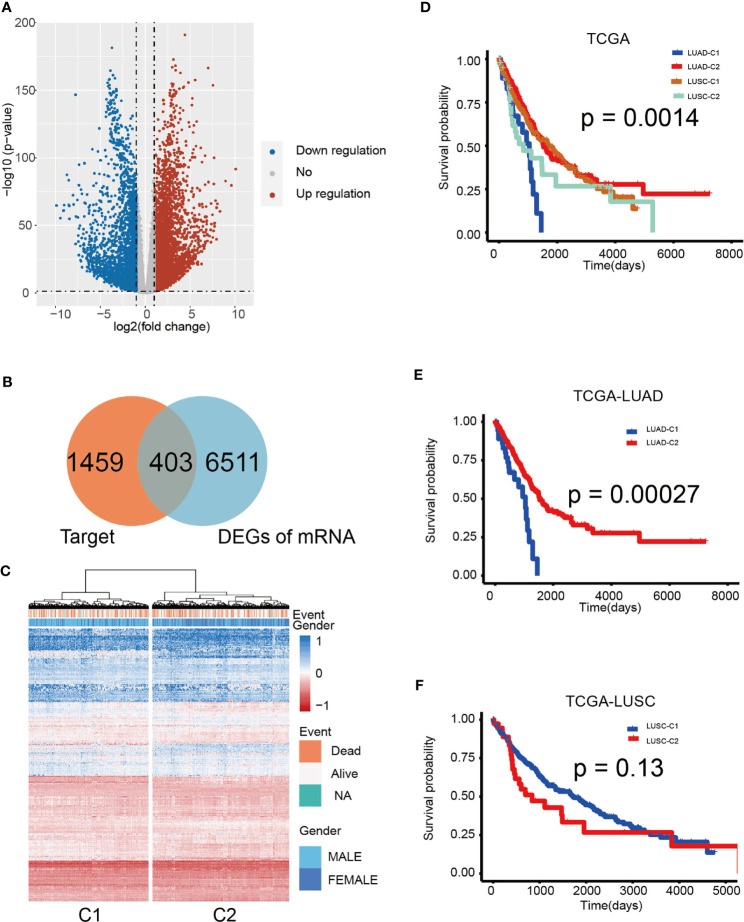
Hierarchical clustering determined distinct sub-clusters linked to variable prognosis based on miRNA targets. **(A)** Volcano plot of 403 identified differential expression miRNAs. Cutoffs were set as log2(fold-change) > 1.0 or < –1.0 and p-value < 0.05. **(B)** Venn diagram shows that the 403 genes are targets and differentially expressed. **(C)** Hierarchical clustering of 1,014 patients from the TCGA cohort using 10 miRNAs and 403 gene expressions. **(D)** Kaplan–Meier curves for cancer-specific survival according to cluster sub-classes. **(E)** Kaplan-Meier analysis of overall survival based on TCGA-LUAD. **(F)** Kaplan-Meier analysis of overall survival according to TCGA-LUSC. The log-rank test was used to perform statistical analysis.

To further investigate the function of the 10 miRNA components in the TIM-Sig, a total of 1,862 experimentally validated targets of the 10-miRNAs signature were extracted from the miRTarBase database (**Figure 5A**). KEGG pathway analysis revealed that the miRNAs were enriched in cancer, transcriptional dysregulation in cancer, the Hippo signaling pathway, cell cycle, the MAPK signaling pathway as well as other cancer signaling pathways (Top-20 results, **Figure 5B**). Moreover, we performed KEGG pathway analysis for the targets of each miRNA and revealed that 8 out of 10 were enriched in cancer and immune-related signaling pathways (**Figure 5C**), suggesting that 10 miRNAs were associated with immune function and metastasis in cancer. Next, to further explore the relationship between NSCLC patient survival and miRNA targets, differentially expressed genes (DEGs) between normal and tumor samples derived from the TCGA dataset were identified. We obtained a total of 6,914 DEGs, of which there were 403 overlaps with 1,862 targets (**Figures 6A, B**). We found that the TCGA cohort could be grouped into two clusters (C1 and C2) by hierarchical clustering using the 403 overlapped genes (**Figure 6C**). Survival analysis showed that LUAD-C2 had a good prognosis (log-rank p = 0.0014; **Figure 6D**). There was marked variability in survival rates in the two groups in the TCGA-LUAD cohort, although none was discovered in the TCGA-LUSC cohort (TCGA-LUAD: log-rank p = 0.00027, TCGA-LUSC: log-rank p = 0.13; **Figures 6E, F**).

### Construction of the TIM-Sig Targets-Based Prognostic Signature

**Figure 7:**
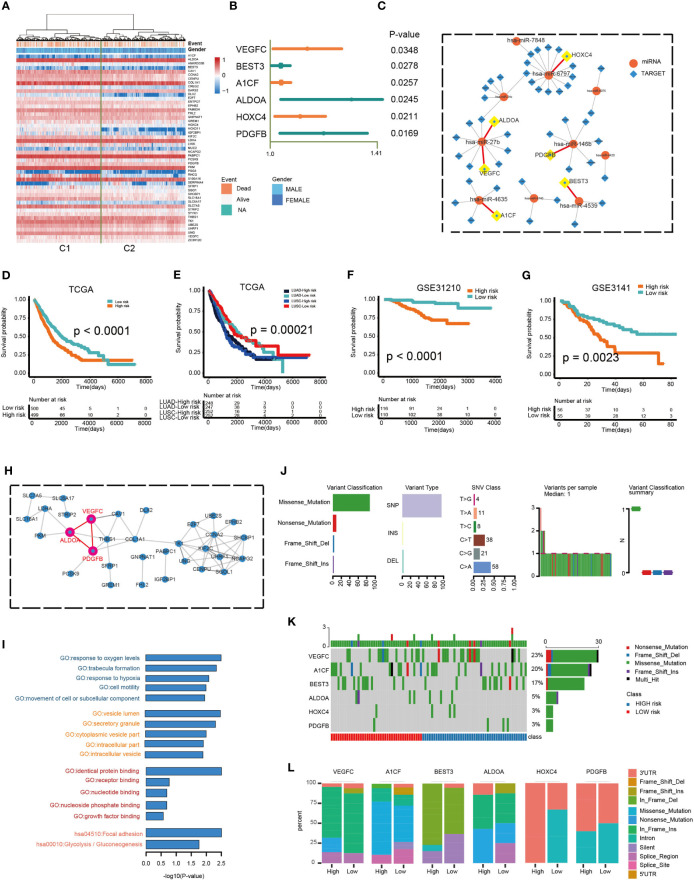
Prognostic potential of a six-gene model. **(A)** Hierarchical clustering of 1,014 patients from the TCGA cohort using 49 gene expressions. **(B)** Multivariate Cox regression analysis was used to determine the prognostic values of DEGs. **(C)** The miRNAs-targets sub-network, includes 49 genes and 10 miRNAs. Circular node denotes miRNAs, square node denotes targets. **(D, E)** Kaplan–Meier predictions of overall survival in patients of high- or low-risk groups as predicted by the RS module in the validation datasets (TCGA and TCGA LUAD/LUSC). **(F, G)** Kaplan–Meier approximation of overall survival in patients of high- or low-risk cohorts as predicted by the RS module in the validation dataset (GSE31210 and GSE3141). The log-rank test was used to perform statistical analysis. **(H)** PPI network. **(I)** Functional enrichment analysis of GO and KEGG for 49 genes. **(J)** Mutation features of 6 risk genes and distribution among high- and low-risk groups. **(K)** The mutation rate of 6 risk genes. **(L)** The difference of mutation location of 6 risk genes between high and low risk groups.

A univariate Cox regression analysis was used to determine the relationship between the 403 overlapped target genes and overall NSCLC patient survival. A total of 49 genes were found to be related to OS in NSCLC patients (**Table S3**). The expression of 49 genes between the C1 and C2 group is shown in **Figure 7A**. **Figure 7C** demonstrates the interaction of miRNAs and the 49 targets. Multivariate Cox regression analysis was then carried out to determine the relationship between genes with OS. Of these, six genes demonstrated significant ability to prognosticate NSCLC (HR > 1, P < 0.05; **Figure 7B** and **Table S4**). The protein-protein interaction (PPI) network also demonstrated close interactions between VEGFC, ALDOA, and PDGFB (**Figure 7H**). The risk scores used for predicting prognostic values were derived as follows: RS (patient) = (0.1190*expression value of VEGFC) + (0.0339*expression value of BEST3) + (0.0351*expression value of A1CF) + (0.2608*expression value of ALDOA) + (0.0960*expression value of HOXC4) + (0.1713*expression value of PDGFB). All subjects were separated into low- or high-risk groups. Kaplan-Meier analysis identified a poorer OS in those of high-risk compared to those of low-risk groups (log-rank p < 0.0001; **Figure 7D**). Prognosis was good in those of LUAD-low-risk and LUSC-low-risk groups (log-rank p = 0.00021; **Figure 7E**). In addition, application of these formulas in our verification cohorts also found that patients with low-risk were more likely to have better OS (GSE31210: log-rank p < 0.0001, GSE3141: log-rank p = 0.0023; **Figures 7F, G**). GO and KEGG pathway analysis revealed that these genes were enriched in cancer and immune-related functions, such as cell motility and Glycolysis/Gluconeogenesis (**Figure 7I**). Finally, we compared the frequency of mutations in the six genes between the two groups (**Figures 7J–L**). The most commonly encountered mutation was the VEGFC mutation (23%, **Figure 7K**). Additionally, we compared the differences of mutation among RS genes in low- and high-risk groups. An obvious difference of mutation location of 6 risk genes between high and low groups were observed. (Wilcoxon test, p < 0.05; **Figure 7L**). Taken as a whole, these findings highlight the significant value of a six-gene signature in prognosticating patients.

## Discussion

A plethora of studies have characterized the immune microenvironment in NSCLC. Li et al. developed a robust, individualized immune signature that can estimate prognosis in patients with early-stage non-squamous NSCLC (32). Hawazin et al. characterized the molecular subtypes of NSCLC, which demonstrated important differences in immune host response (33). Also, our previous study identified different molecular subtypes of NSCLC according to the immune landscape and constructed a prognostic model (18). However, the role of non-coding RNA, especially miRNAs in the NSCLC immune microenvironment have not been well elucidated. Some immune-related miRNAs were found to be fundamental in the regulation of innate and immune responses to tumor cells. But these studies were only focused on individual immune-related miRNAs in limited samples. In this study, we identified potential tumor immune-related miRNA biomarkers from miRNA-seq profiling data in TCGA. The constructed immune-related miRNA signature was tested and found to be able to function as a means stratify the risk of NSCLC patients. Of these 10 miRNAs included in the signature, a number have previously been explored in cancer research. One example is the central role of miR-146a in the melanoma immune microenvironment (34). Combined inhibition of PD-1 and miR-146a may be able to elicit an anti-tumor immune response (34). Furthermore, miR-27b has been characterized as a biomarker for recurrent ovarian cancer (35). Our novel miRNA profile also includes yet to be reported miRNAs which may hold significant prognostic values in NSCLC. The immune-associated functions of these miRNAs were confirmed by stratifying the subjects into low- and high-risk cohorts. We found that the low-risk group had a markedly higher immune score and lower tumor purity. These observations were confirmed by the higher tumor cell aggregation in the low-risk group which was represented by TILs and TIS. This is consistent with previous reports that a high degree of immune cell infiltration was responsible for a significantly favorable prognosis in NSCLC (18). Our results also demonstrated that the low-risk group possessed raised CYT and TMB expressions, with higher degrees of CD8+ T cell infiltration. Similar findings of better outcomes in those with higher CYT levels have also been reported in cancer patients (18, 36). In patients with resected NSCLC, higher TMB scores were indicative of a more favorable prognosis (37). CD8+ T cell infiltration appeared to function as a superior predictive biomarker in response to anti-PD-1 immunotherapy (38).

Our data indicated that the TIM-Sig score was significantly higher in TP53 and CSMD3 mutation samples. Other studies have reported a higher proportion of activated immune cell infiltration in patients with TP53 mutations, resulting in a significantly prolonged progression-free survival in the LUAD cohort (39). CSMD3 mutations have been characterized as tumors with high concentrations of T cells in patients with high-grade serous ovarian carcinoma (40). In addition, the CSMD3 mutation was related to improved response to anti-PD1/PD-L1 and higher survival rates solid tumors (41, 42).

Previous studies have shown that immunogenomic-derived immune scores were indicative of chemotherapeutic benefits (43). We subsequently investigated whether the TIM-Sig could predict chemosensitivity in NSCLC. Our results suggested that the IC50 values were significantly higher in the low-risk group for some anti-cancer agents. Among these agents, irinotecan represents a widely used chemotherapeutic medication in treating solid tumors and its sensitivity has been reported to correlate with CD8+ T cell fraction in pancreatic cancer (44). CD8 effector cells have previously been reported to enhance the anti-tumor response of methotrexate, another anti-cancer agent, in breast cancer (45). However, we are unable to investigate TIM-Sig prognostic significance in regard to response to immunotherapy due to the lack of subjects who received treatment involving immune checkpoint inhibitors. This is one limitation in our study.

Functional analysis of these 10 miRNAs in TIM-Sig may further our understanding of their individual roles in NSCLC. To this end, we obtained the validated targets of these miRNAs. KEGG pathway analysis uncovered that the target genes were most enriched in cancer-related pathways. Additionally, the targets of individual miRNAs were also enriched in cancer and immune-related pathways comprising of pathways in cancer, adherens junction, the chemokine signaling pathway, and the HIF-1 signaling pathway. Dysregulation of adherens junction function is critical in modulating efficient collective invasion and migration of carcinoma cells (46). The adherens junction was also an activated pathway in breast cancer cases with low immunity (29). The classification based on HTF 1 signaling pathway profile was able to determine subgroups of prostate cancer patients who were maximally responsive to chemo- and immunotherapy (47). The components of our constructed 10-miRNA signature were strongly involved in immune function and cancer metastasis.

Next, we identified 403 differentially expressed target genes between normal lung and NSCLC samples. The expression profile of these genes revealed two distinct sample clusters with different outcomes. The two patient clusters in the TCGA-LUAD cohort had significantly different survival outcomes. However, no significant difference was observed in the TCGA-LUSC cohort. Although LUAD and LUSC are the most frequently encountered NSCLC subtypes, they vary from each other considerably (48, 49). LUSC has been found to grow at a faster rate in contrast to LUAD. LUSC was also found to possess suppressed expressions of molecules involved in the activation of the immune response, such as chemokines and MHC molecules (50). These findings might explain the different results of survival analysis between LUAD and LUSC. In efforts to improve the prognostic performance of the target genes, we identified six differentially expressed target genes which were correlated with survival: VEGFC, ALDOA, BEST3, A1CF, HOXC4, and PDGFB. Subjects in the TCGA cohort were stratified into high- or low-risk groups using the gene-based RS. Low-risk groups of both LUAD and LUSC had significantly better survival than those in the high-risk groups. Among these six genes, VEGFC, ALDOA, and PDGFB closely interacted with each other in the PPI network. It has been reported that VEGFC knockdown results in reduced PDGFB levels in melanoma cell lines. Moreover, both of them were regulated by E2F1 in angiogenesis (51). Lung cancer metastasis and metabolic reprogramming appears to be strongly dependent on ALDOA (52). Samples of lung cancer have been noted to possess an overexpression of ALDOA, which enhances epithelial-mesenchymal transition (53). Our data suggested that immune-related miRNAs regulated immune cell infiltration in NSCLC both through themselves and their target genes. In summary, our study on the identification of tumor immune-associated miRNAs provides valuable functional insights and potential clinical guidance for personalized therapy for NSCLC patients.

## Conclusions

In brief, this study aimed to construct an immune-related miRNA signature that effectively predicts NSCLC patient prognosis. An immune-related miRNA signature (TIM-Sig) was constructed using LASSO-penalized multivariate survival models and was evaluated in several public cohorts from the Gene Expression Omnibus (GEO) and the CellMiner database. Further analysis on the miRNA-target network of TIM-Sig revealed that these target genes had prognostic value in both lung squamous cell carcinoma and adenocarcinoma. Our study provides valuable functional insights and potential clinical guidance for personalized therapy for NSCLC patients.

## Data Availability Statement

The original contributions presented in the study are included in the article/**Supplementary Material**, further inquiries can be directed to the corresponding authors.

## Author Contributions

SJ and LY designed the study. YZ, ZL, LQ, and ML collected data. YZ and KM developed the computational model and analyzed the data. SJ and YZ wrote the article. All authors reviewed the manuscript. All authors contributed to the article and approved the submitted version.

## Funding

This work was supported by the National Natural Science Foundation of China (81670028), the Major Program of Natural Science Foundation of Heilongjiang Province (ZD2016014), and the Harbin City Applied Technology Research and Development Project (excellent subject leader) (2016RQXYJ116).

## Conflict of Interest

The authors declare that the research was conducted in the absence of any commercial or financial relationships that could be construed as a potential conflict of interest.

## Supplementary Material

Supplementary Figure 1 Strategy for determining tumor immune infiltration-related miRNA signature (TIM-Sig) in this study.

Supplementary Figure 2 Expression of 78 miRNAs and immune score. **(A)** The expression profile of 78 miRNAs landscape in the TCGA cohort. **(B, C)** MiRNAs expression associated with stromal score and tumor purity, as shown by Spearman correlation analysis.

Supplementary Figure 3 The difference of proportion of gender, stage and TNM in high- and low-risk cohorts.

Supplementary Figure 4
**(A–J)** Immunization-related indicators could describe the significant relationship between the key biomarkers and TIM-Sig.

Supplementary Figure 5 The difference of mutation type of top 5 mutation genes in high- and low-risk cohorts.

## Abbreviations

NSCLC, non-small cell lung cancer; miRNAs, microRNAs; TCGA, The Cancer Genome Atlas; LASSO, least absolute shrinkage and selection operator; TIM-Sig, tumor immune-related miRNA signature; GEO, Gene Expression Omnibus; OS, overall survival; TMB, tumor mutation burden; CYT, cytotoxic activity; TIS, T cell infiltration score; APM, antigen presentation machinery; TILs, tumor-infiltrating lymphocytes; MHC, major histocompatibility complex; LUAD, lung adenocarcinoma; LUSC, lung squamous cell carcinoma.
