# Supplementary figures and images for: Identification of Prognostic miRNAs Associated With Immune Cell Tumor Infiltration Predictive of Clinical Outcomes in Patients With Non-Small Cell Lung Cancer
Source: Front Oncol. 2021 Jul 1;11:705869. doi: 10.3389/fonc.2021.705869 (PMC8281680; doi:10.3389/fonc.2021.705869)

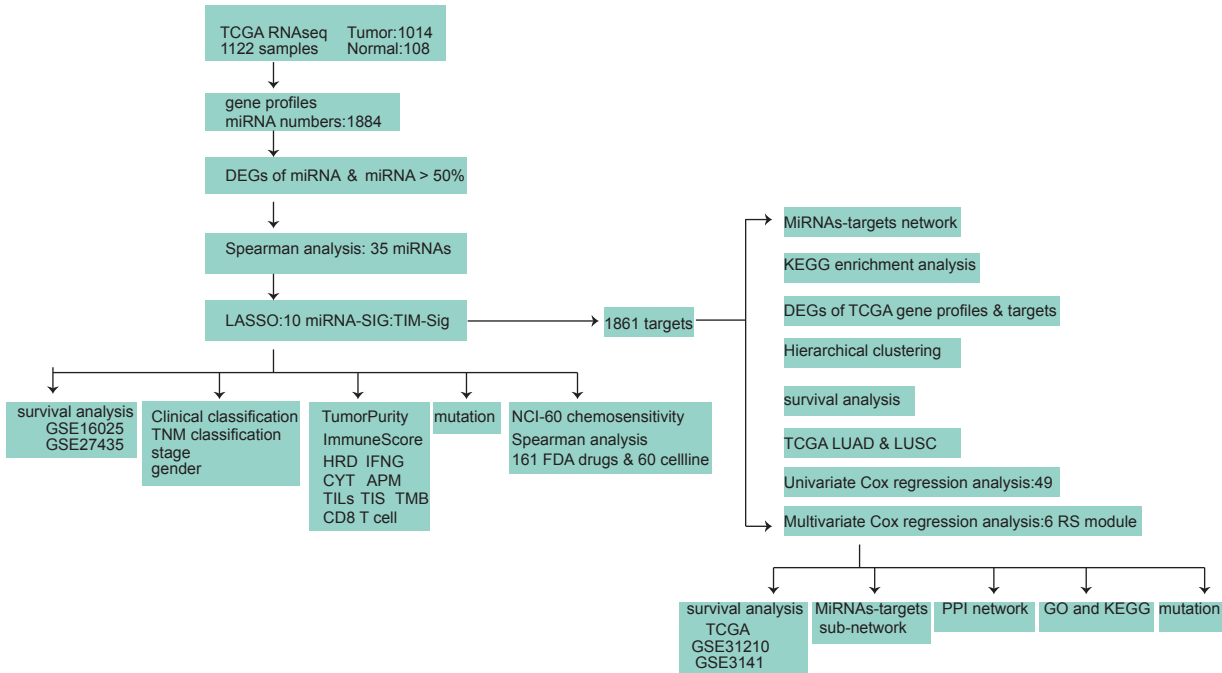

Supplement: Supplementary Figure 1 — Strategy for determining tumor immune infiltration-related miRNA signature (TIM-Sig) in this study. [file Image_1.pdf]

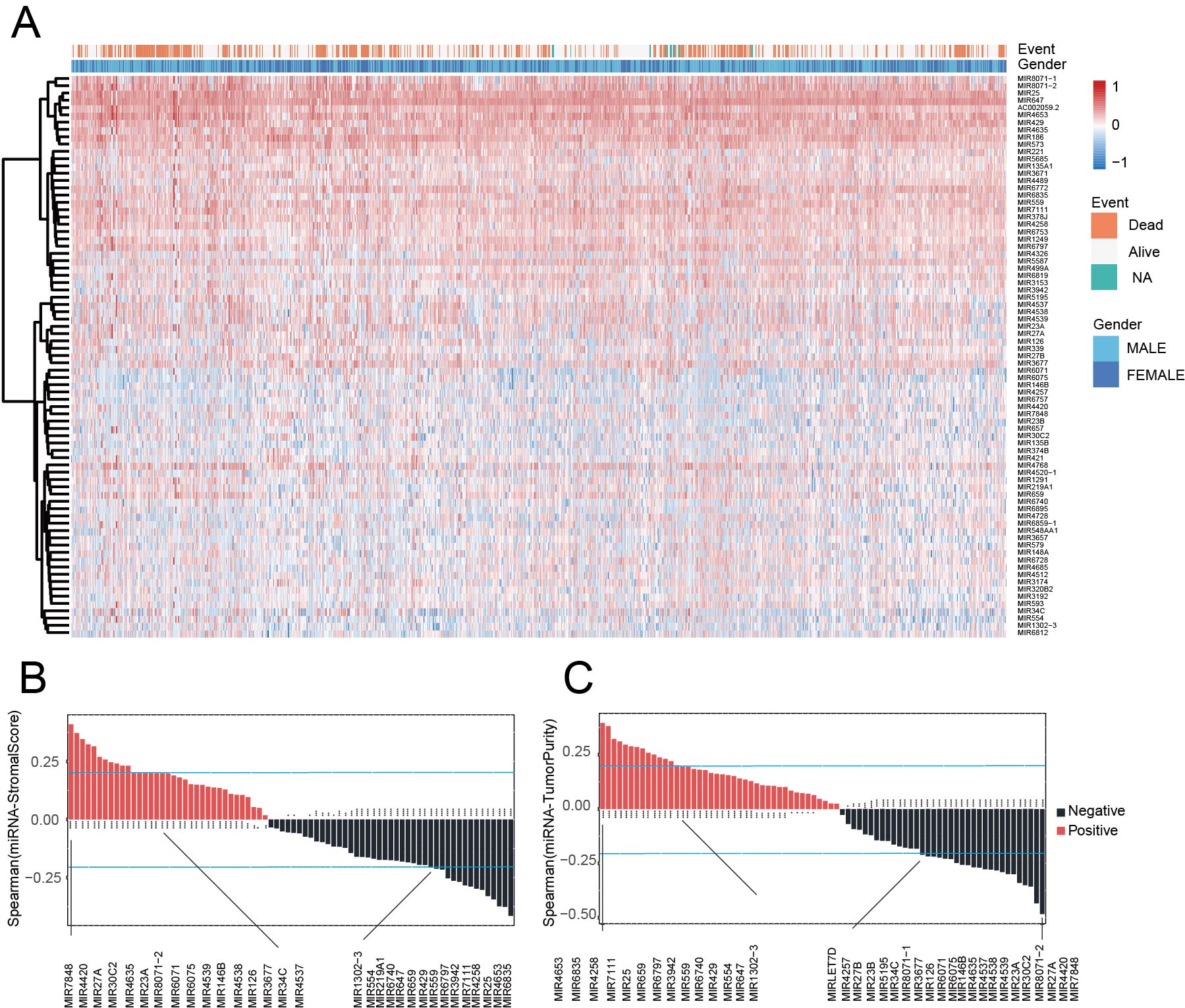

Supplement: Supplementary Figure 2 — Expression of 78 miRNAs and immune score. (A) The expression profile of 78 miRNAs landscape in the TCGA cohort. (B, C) MiRNAs expression associated with stromal score and tumor purity, as shown by Spearman correlation analysis. [file Image_2.jpg]

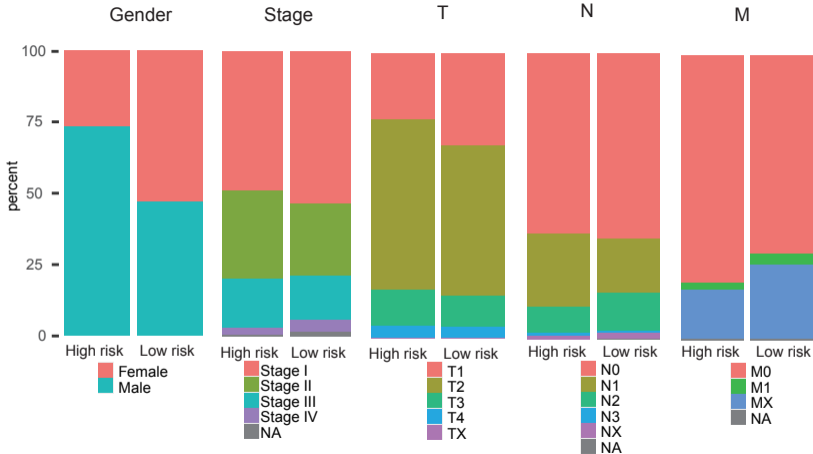

Supplement: Supplementary Figure 3 — The difference of proportion of gender, stage and TNM in high- and low-risk cohorts. [file Image_3.pdf]

**A**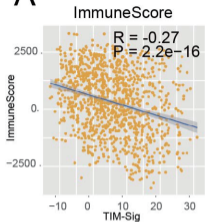**B**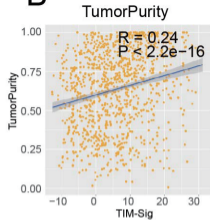**C**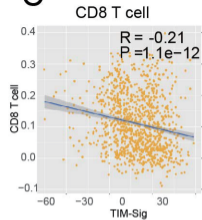**D**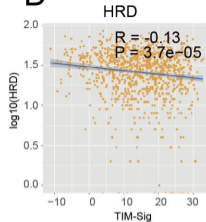**E**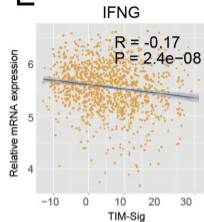**F**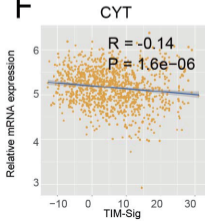**G**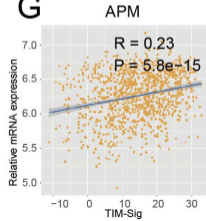**H**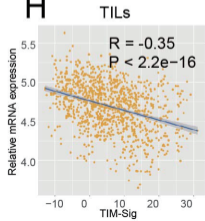**I**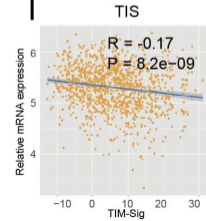**J**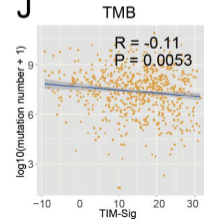

Supplement: Supplementary Figure 4 — (A–J) Immunization-related indicators could describe the significant relationship between the key biomarkers and TIM-Sig. [file Image_4.pdf]

TP53

TTN

MUC16

CSMD3

RYR2

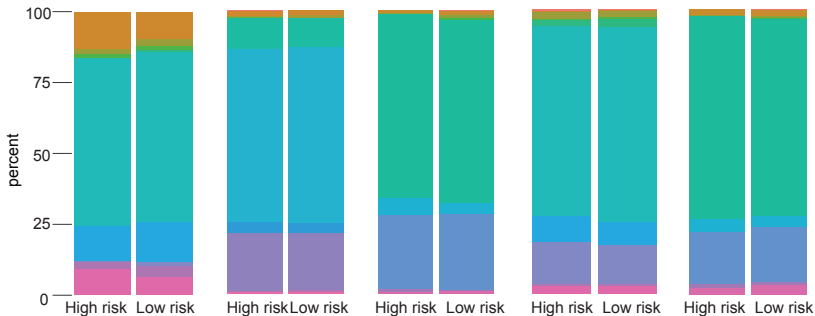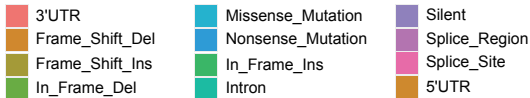

Supplement: Supplementary Figure 5 — The difference of mutation type of top 5 mutation genes in high- and low-risk cohorts. [file Image_5.pdf]
